# Supplementary material for: Clinical and Molecular Characteristics of KRAS Codon-Specific Mutations in Advanced Pancreatic Ductal Adenocarcinoma with Prognostic and Therapeutic Implications
Source: Int J Mol Sci. 2025 Nov 11;26(22):10908. doi: 10.3390/ijms262210908 (PMC12652077; doi:10.3390/ijms262210908)
Supplement: Supplementary file 1 [file ijms-26-10908-s001.zip › ijms-3903719-supplementary.pdf]

Supplementary Materials

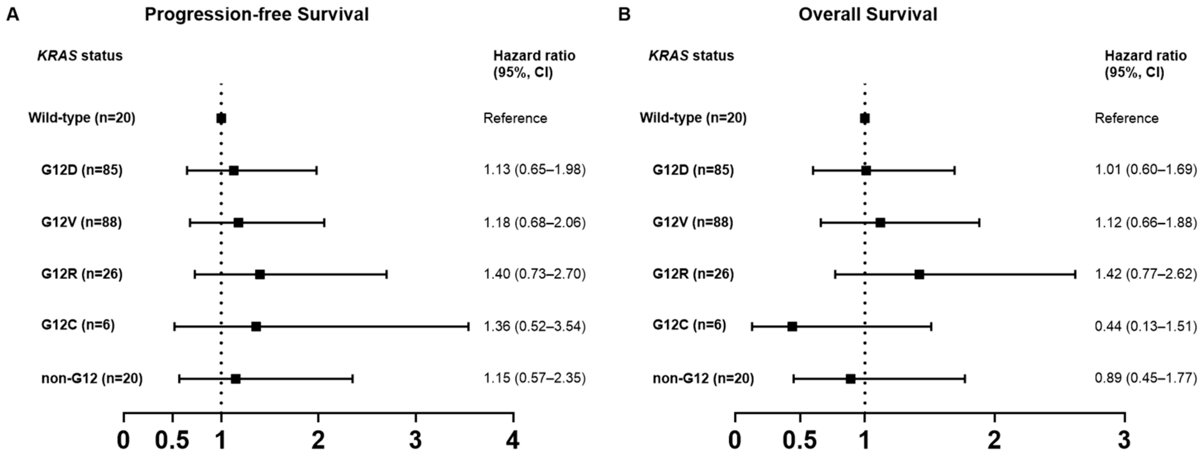

Figure S1. Comparison of median progression-free survival (A) and overall survival (B) between *KRAS* wild-type and *KRAS* codon-specific subtypes in the treated cohort.

Table S1. Actionable genomic alterations identified in *KRAS* wild-type pancreatic cancer patients

**Wild type (n = 22)**	**Gene alterations**	**Variant type**
Patient 1	*BRAF* Thr599dup, *TP53*, *ARID1A*	SNV
Patient 2	*AR*, *ARAF* amplification	CNV
Patient 3	*BRCA2*, *TP53*	SNV
Patient 4	*MDM2* amplification	CNV
Patient 5	*TP53*, *CDKN2A*	SNV
Patient 6	*EGFR* L858R, *PIK3CA* H1047R	SNV
Patient 7	*EGFR* exon 19 deletion, *ATM*	SNV
Patient 8	*AR*, *ARAF* amplification	CNV
Patient 9	Not detected	
Patient 10	*BRAF* V600E	SNV
Patient 11	*BRAF* V600E	SNV
Patient 12	*PTEN*, *RNF43*, *SND1-BRAF* fusion	SNV, Fusion
Patient 13	*PIK3CA* P539R, *SMAD4*	SNV
Patient 14	Not detected	
Patient 15	*ARID1A*	SNV
Patient 16	*BRAF V600E*, *TP53*	SNV
Patient 17	*BAP1*, *FGF3*, *PIK3R1*	SNV
Patient 18	*TP53*, *CDKN2A*, *NF1*	SNV
Patient 19	*TP53*, *PIK3CA* H1047R	SNV
Patient 20	Not detected	
Patient 21	*CDKN2A*	SNV
Patient 22	*TP53*	SNV
***BRAF* V600E**	3 (13.6%)	
***EGFR* mutation**	2 (9.1%)	
***PIK3CA* mutations**	3 (3.6%)	

SNV single nucleotide variant, *CNV* copy number variation.

Table S2. Comparison of clinical characteristics according to *KRAS* mutation subtypes in patients with pancreatic cancer

Variable	G12D (n = 93)	G12V (n = 97)	G12R (n = 28)	Non-G12 (n = 21)	Wild type (n = 22)	*p* value
**Age, years**						
< 65	43 (46.2)	37 (38.1)	9 (32.1)	7 (33.3)	7 (31.8)	0.501
≥ 65	50 (53.8)	60 (61.9)	19 (67.9)	14 (66.7)	15 (68.2)	
**Gender**						
Male	50 (53.8)	54 (55.7)	11 (39.3)	11 (52.4)	11 (50.0)	0.651
Female	43 (46.2)	43 (44.3)	17 (60.7)	10 (47.6)	11 (50.0)	
**Tumor location**						
Head	49 (52.7)	48 (49.5)	11 (39.3)	9 (42.9)	14 (63.6)	0.459
Body/Tail	44 (47.3)	49 (50.5)	17 (60.7)	12 (57.1)	8 (36.4)	
**Differentiation**						
Well to moderately	52 (70.3)	52 (70.3)	17 (73.9)	14 (82.4)	12 (80.0)	0.836
Poorly	22 (29.7)	18 (25.4)	6 (26.1)	3 (17.6)	3 (20.0)	
**No. of organ metastasis**						
0-1	62 (66.7)	68 (70.1)	17 (60.7)	15 (71.4)	15 (68.2)	0.900
≥ 2	31 (33.3)	29 (29.9)	11 (39.3)	6 (28.6)	7 (31.8)	
**Site of metastasis**						
Liver	55 (59.1)	48 (49.5)	15 (53.6)	12 (57.1)	10 (45.5)	0.644
Lung	12 (12.9)	19 (19.6)	8 (28.6)	4 (19.0)	2 (9.1)	0.268
Peritoneum	21 (22.6)	27 (27.8)	9 (32.1)	4 (19.0)	7 (31.8)	0.700
**CA 19-9**						
Normal	20 (22.2)	14 (14.6)	4 (14.8)	8 (42.1)	9 (40.9)	0.011
Elevated (≥ 34U/mL)	70 (77.8)	82 (85.4)	23 (85.2)	11 (57.9)	13 (59.1)	
**CEA**						
Normal	47 (52.8)	49 (52.1)	17 (63.0)	7 (36.8)	14 (63.6)	0.395
Elevated (≥ 5.0 μg/L)	42 (47.2)	45 (47.9)	10 (37.0)	12 (63.2)	8 (36.4)	
**NLR**						
< 3.0	60 (66.7)	56 (57.7)	16 (59.3)	8 (40.0)	11 (50.0)	0.201
≥ 3.0	30 (33.3)	41 (42.3)	11 (40.7)	12 (60.0)	11 (50.0)	

CA 19-9 carbohydrate antigen 19-9, *CEA* carcinoembryonic antigen, *NLR* neutrophil to lymphocyte ratio. Data are n (%).

Table S3. Treatment outcomes by first-line chemotherapy regimen in the treated cohort and *KRAS* G12D/G12V subgroups

	Total (n = 245)			G12D (n =85)			G12V (n = 88)		
**Variable**	**Gem-based (n = 138)**	**5-FU based (n = 107)**	***p* value**	**Gem-based (n = 138)**	**5-FU based (n = 107)**	***p* value**	**Gem-based (n = 138)**	**5-FU based (n = 107)**	***p* value**
**Objective response rate**	52 (37.7)	35 (32.7)	0.50	21 (42.9)	12 (33.3)	0.50	15 (30.6)	15 (38.5)	0.50
**Disease control rate**	117 (84.8)	92 (86.0)	0.86	44 (89.8)	31 (86.1)	0.74	39 (79.6)	36 (92.3)	0.13
**mPFS, months [95% CI]**	5.8 [5.3–7.2]	7.8 [5.7–9.6]	0.12	5.8 [5.4–7.5]	6.9 [4.8–11.8]	0.45	5.3 [3.7–7.6]	8.3 [6.9–12.8]	0.01
**6-months PFS rate, %**	48.4 [40.5–57.9]	56.0 [47.2–66.5]		48.3 [35.8–65.3	50.0 [36.1–69.3]		41.6 [29.4–58.8]	68.0 [54.6–84.8]	
**mOS, months, [95% CI]**	9.7 [8.0–12.6]	13.1 [10.8–16.0]	0.08	10.5 [8.3–17.2]	13.1 [8.8–19.6]	0.93	6.6 [4.9–13.0]	14.8 [13.0–19.3]	0.01
**12-months OS rate, %**	41.4 [33.8–50.8]	56.3 [47.5–66.8]		47.7 [35.5–64.3]	57.6 [43.4–76.5]		36.6 [25.0–53.4]	66.4 [53.0–83.1]	

Gem-based gemcitabine-based; *5-FU-based* fluorouracil-based; m*PFS* median progression-free survival; m*OS* median overall survival. Data are n (%). PFS and OS rates are expressed as percentages with 95% confidence intervals.
